# Physician Transfer Versus Patient Transfer for Mechanical Thrombectomy in Patients With Acute Ischemic Stroke: A Systematic Review and Meta‐Analysis

**DOI:** 10.1161/JAHA.123.031906

**Published:** 2024-06-20

**Authors:** Adnan I. Qureshi, Abdullah Lodhi, Hamza Maqsood, Xiaoyu Ma, Gordian J. Hubert, Camilo R. Gomez, Chun S. Kwok, Daniel E. Ford, Daniel F. Hanley, David R. Mehr, Qaisar A. Shah, M. Fareed K. Suri

**Affiliations:** ^1^ Zeenat Qureshi Stroke Institutes St Cloud MN USA; ^2^ Department of Neurology University of Missouri Columbia MO USA; ^3^ Department of Neurology, TEMPiS Telestroke Center München Klinik gGmbH Munich Germany; ^4^ Department of Cardiology, Queen Elizabeth Hospital Birmingham University Hospitals of Birmingham NHS Trust Stoke‐on‐Trent UK; ^5^ Department of Medicine Johns Hopkins University Baltimore MD USA; ^6^ Department of Neurology Johns Hopkins University Baltimore MD USA; ^7^ Department of Geriatric Medicine University of Missouri Columbia MO USA; ^8^ Department of Neurology Winchester Medical Center Winchester VA USA; ^9^ Department of Neurology CentraCare St Cloud MN USA

**Keywords:** functional independence, mechanical thrombectomy, patient transfer, physician transfer, time intervals, Cerebrovascular Disease/Stroke

## Abstract

**Background:**

Physician transfer is an alternate option to patient transfer for expedient performance of mechanical thrombectomy in patients with acute ischemic stroke.

**Methods and Results:**

We conducted a systematic review to identify studies that evaluate the effect of physician transfer in patients with acute ischemic stroke who undergo mechanical thrombectomy. A search of PubMed, Scopus, and Web of Science was undertaken, and data were extracted. A statistical pooling with random‐effects meta‐analysis was performed to examine the odds of reduced time interval between stroke onset and recanalization, functional independence, death, and angiographic recanalization. A total of 12 studies (11 nonrandomized observational studies and 1 nonrandomized controlled trial) were included, with a total of 1894 patients. Physician transfer was associated with a significantly shorter time interval between stroke onset and recanalization with a pooled mean difference estimate of −62.08 (95% CI, −112.56 to −11.61]; *P*=0.016; 8 studies involving 1419 patients) with high between‐study heterogeneity in the estimates (*I* ^2^=90.6%). The odds for functional independence at 90 days were significantly higher (odds ratio, 1.29 [95% CI, 1.00–1.66]; *P*=0.046; 7 studies with 1222 patients) with physician transfer with low between‐study heterogeneity (*I* ^2^=0%). Physician transfer was not associated with higher odds of near‐complete or complete angiographic recanalization (odds ratio, 1.18 [95% CI, 0.89–1.57; *P*=0.25; *I* ^2^=2.8%; 11 studies with 1856 subjects).

**Conclusions:**

Physician transfer was associated with a significant reduction in the mean of time interval between symptom onset and recanalization and increased odds for functional independence at 90 days with physician transfer compared with patient transfer among patients who undergo mechanical thrombectomy.

**Nonstandard Abbreviations and Acronyms**
CSC: comprehensive stroke center
ESCAPE: Endovascular Treatment for Small Core and Proximal Occlusion Ischemic Stroke
MT: mechanical thrombectomy
NOS: Newcastle–Ottawa Scale
sICH: symptomatic intracranial hemorrhage

**Clinical Perspective**

**What Is New?**

- Transferring the neurointerventionalist to the hospital at which the patient presents with acute ischemic stroke is associated with decreased time interval between symptom onset and recanalization.

**What Are the Clinical Implications?**

- Physician transfer results in increased odds of functional independence at 90 days in patients who undergo mechanical thrombectomy for acute ischemic stroke.
- It may create a more favorable ratio between number of neurointerventionalists and procedural volume for each neurointerventionalist.

Regional customization of stroke systems of care is required to address differences in resources, hospital certifications, geography, and population density, which is important to maximize treatment opportunities for patients eligible for mechanical thrombectomy (MT).^1^ One aspect of stroke systems of care is to evaluate various approaches to delivering MT so that the access to this treatment could be maximized among eligible patients with acute ischemic stroke.^1^ There are 2 potential methods of providing MT to patients with acute ischemic stroke: transferring the neurointerventionalist to the hospital at which the patient presents with acute ischemic stroke (physician transfer) or transferring the patient from the initial hospital to a hospital that performs MT (patient transfer).^2^ A few studies with small sample sizes have suggested that physician transfer can reduce the time from emergency department arrival to initiation of MT^3^, ^4^, ^5^, ^6^, ^7^ and increase the rates of functional independence after the procedure.^3^, ^5^, ^6^, ^8^, ^9^, ^10^, ^11^, ^12^, ^13^, ^14^ Due to the small number of patients in individual studies, we performed a meta‐analysis to systematically assess the existing literature and pool together the studies to estimate the effect of physician transfer compared with patient transfer.

## Methods

The details of our systematic review were uploaded for registration on the Prospective Register of Systematic Reviews, which can be accessed at https://www.crd.york.ac.uk/prospero/#recordDetails with ID: CRD42023444143. The authors declare that all supporting data are available within the article. The protocol for systematic review was reviewed and approved by the institutional review board (MU Institutional Review Board No. 2100666). The requirement for informed consent was waived by the institutional review board.

### Literature Search Strategy

This systematic review and meta‐analysis were conducted and reported according to the Preferred Reporting Items for Systematic Reviews and Meta‐Analyses guidelines.^15^ We have completed the Preferred Reporting Items for Systematic Reviews and Meta‐Analyses checklist. Two reviewers (A.L. and H.M.) searched the online databases of PubMed, Scopus, Embase, and Web of Science (from January 2012 until March 2023) to find all relevant observational studies, clinical trials, clinical studies, comparative studies, and multicenter studies. We also searched clinicaltrials.gov to identify any additional articles. We started the search from January 2012 to focus on studies that used contemporary MT devices, which were initially approved in 2012^11^ (see Data S1 for detailed search strategy). Studies in non‐English language were excluded. Preprint repositories (ie, Research Square and MedRxiv) were searched for any preprint or unpublished manuscripts. Relevant studies identified during the screening process then entered full‐text review, in which full‐text manuscripts were evaluated independently by reviewers (A.L. and H.M.) for eligibility and reference lists from these studies to identify additional eligible studies. Any uncertainties about including a study in the review were discussed with the study's supervisor (A.I.Q.) for resolution.

### Eligibility Criteria

Studies were included if they comprised adult patients aged ≥18 years with acute ischemic stroke due to large‐vessel occlusion in the anterior or posterior circulation. Data also had to be available for 2 arms: (1) transferring a neurointerventionalist from the thrombectomy providing center to a non–thrombectomy‐providing hospital for thrombectomy (physician transfer) and (2) transferring patients from a non–thrombectomy‐providing hospital to a thrombectomy‐providing center for thrombectomy (patient transfer). MT included aspiration techniques, stent retrievers, stent placement, and balloon angioplasty. We excluded studies with essentially the same data sets or no specific data regarding patients receiving thrombectomy in either group. In addition, the included studies had to report outcomes for the physician transfer and patient transfer groups. The primary outcome of our study was the time interval from symptom onset to recanalization. If time is given in median (±interquartile range) in the included studies, we converted the value to mean (±SD) by assuming mean=median and SD=interquartile range/1.35.^16^ For studies that reported sequence of events, we added the mean time together and added SD by total SD=sqrt (SD1^2^+SD2^2^). Secondary outcomes were functional independence at 90 days (modified Rankin Scale score, 0–2), all‐cause death within 90 days, occurrence of post‐MT symptomatic intracranial hemorrhage (sICH) as defined by each study, and successful recanalization on angiogram (Thrombolysis in Cerebral Infarction/Thrombolysis in Myocardial Infarction score of ≥2b).

### Data Extraction

Two reviewers (A.L. and H.M.) independently extracted study ID, author, year, characteristics, and quality data from the included studies. The study characteristics that were extracted included: (1) country of origin; (2) traveling time and distance from comprehensive stroke center (CSC) to primary stroke center; (3) mode of physician transfer; (4) mode of patient transfer; (5) location of occlusion; (6) comparison (either the cases and controls of studies included are matched, unmatched, or randomized); and (7) design of trial (prospective cohort or retrospective cohort or nonrandomized controlled study). Primary and secondary outcomes data and the sICH definitions applied in each study were also extracted.

### Risk‐of‐Bias Assessment in Studies

The quality of the included 11 observational studies^3^, ^4^, ^5^, ^6^, ^7^, ^8^, ^9^, ^10^, ^12^, ^13^, ^14^, ^15^ was assessed by 2 independent reviewers (A.L. and H.M.) for risk of bias using the Newcastle–Ottawa Scale (NOS) for cohort studies.^17^ The NOS evaluates 3 quality parameters (selection, comparability, and outcome), with a maximum of 4 points for selection, 2 points for comparability, and 3 points for outcomes, with a maximum total score of 9 for each study, representing a good‐quality study.^17^ Studies having a total score of <5 are identified as studies with a high risk of bias. For assessing the quality of the 1 nonrandomized controlled study,^11^ we used a methodological index for nonrandomized studies, which assess quality on a 12‐item scale with every item use: not reported (0 point), reported but inadequate (1 point), or reported and adequate (2 points) to judge.^18^

### Statistical Analysis

We calculated the standardized mean difference for the outcome of time from stroke onset to recanalization. For the outcomes of functional independence at 90 days, all‐cause death within 90 days, the occurrence of post‐MT sICH as defined by each study, and successful recanalization on angiogram, we calculated odds ratios (ORs) as effect size for each comparison. Random‐effects models were used to account for heterogeneity and generate overall *P* values for assessing statistical significance. The heterogeneity was described with the *I* ^2^ statistic. Meta‐regression was used to explore the heterogeneity attributed to (1) country of origin; (2) mode of physician transfer (by air versus by ground); (3) design of trial (prospective cohort or retrospective cohort or nonrandomized controlled study); (4) comparison (either the cases and control of studies included are unmatched, matched, or randomized); and (5) study quality. Funnel plots were used to test the presence and extent of publication bias. Adjusted effect sizes were estimated by incorporating theoretically missing studies using the trim‐and‐fill approach.^19^ There was no difference between the direction and magnitude of association in fixed‐effect models (data not shown) and random‐effect models; therefore, only the results from the random‐effect model are presented. Sensitivity analyses were performed for the primary outcome only (time interval from stroke onset to recanalization) considering (1) repeating the analysis by including the studies with high‐quality scores (>8) and (2) repeating the analysis by including the studies that used unmatched comparators. We also calculated the median difference for the outcome of time from stroke onset to recanalization and performed the meta‐analysis accordingly.^20^ Due to the small number of events in some of the studies in the analyses for the outcomes of death and sICH, we performed a sensitivity analysis using a leave‐one‐out meta‐analysis.^21^ Statistical analyses were performed using R version 4.2.0, R package meta version 5.2.0, and metamedian version 1.1.0 (R Foundation for Statistical Computing, Vienna, Austria).^22^ All estimates included a 95% CI, and *P* values <0.05 were considered statistically significant.

## Results

### Literature Search and Study Characteristics

**Figure 1:**
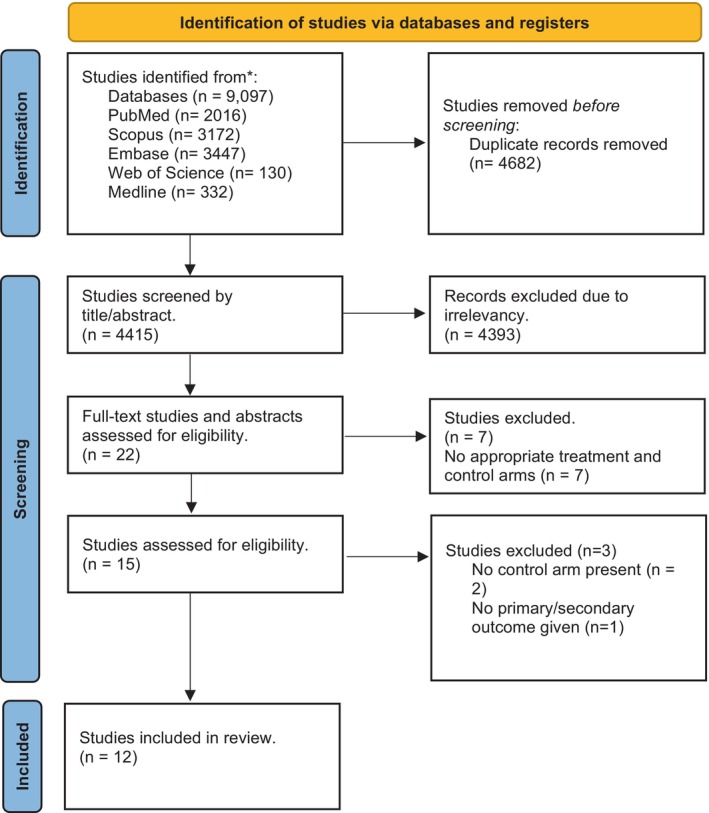
Flowchart depicting identification of studies through literature search.

A flow diagram depicting the study selection for physician transport versus patient transport is shown in Figure 1. A total of 9097 potentially relevant studies were identified in the search. A total of 12 studies (11 nonrandomized observational studies and 1 nonrandomized controlled trial) met the inclusion criteria. It is notable that 2 studies were excluded because they had no control group.^23^, ^24^ Another study was excluded because it did not have primary or secondary outcomes.^25^ A summary of study characteristics of included studies is shown in the Table1. Definitions of sICH and successful reperfusion of included studies are shown in Table S1. Six studies were from Germany, 3 from the United States, 2 from China, and 1 from Spain. The mode of physician transport was air and ground in 2^4^, ^11^ and 10 studies,^3^, ^5^, ^6^, ^7^, ^8^, ^9^, ^10^, ^12^, ^13^, ^14^ respectively.

**Table 1:** Study Characteristics of the Included Studies

Author	Country of origin	Mode of physician transported	Design of trial (prospective, retrospective)	Comparison (unmatched, matched, randomized)	Distance and time from comprehensive stroke center	Mode of patient transfer	Location of occlusion: anterior circulation (ICA, MCA); posterior circulation (BA, VA)
Hou et al, 2022^3^	China	By ground	Prospective cohort study	Unmatched	Not available	Not available	ICA, MCA, vertebrobasilar artery
Kettner et al, 2023^4^	Germany	By air	Prospective cohort study	Unmatched	Flying distance was 50 km, while driving distance was 36 km	Emergency ambulance	ICA, MCA BA, VA, ACA
Morey et al, 2020^5^	United States	By ground	Prospective cohort study	Matched	Location within 32 km	Emergency ambulance	ICA, MCA
Morey et al, 2021^6^	United States	By ground	Prospective cohort study	Matched	Location within 32 km	Emergency ambulance	ICA, MCA
Wei et al, 2017^7^	United States	By ground	Retrospective cohort study	Unmatched	Location within 32 km or transport time of max 1 h from CSC	Emergency ambulance	ICA, MCA
Hang et al, 2022^8^	China	By ground	Retrospective cohort study	Unmatched	Location within 50 km or transport time within 1 h from CSC	Emergency ambulance	ICA, MCA
Seker et al, 2021^9^	Germany	By ground	Prospective cohort study	Unmatched	Not available	Not available	ICA, MCA
Seker et al, 2018^10^	Germany	By ground	Prospective cohort study	Unmatched	Location within 100 km or transport time within 1 h from CSC	Emergency ambulance	ICA, MCA
Hubert et al, 2022^11^	Germany	By air	Nonrandomized controlled study	Unmatched	Not available	Helicopter or emergency ambulance	ICA, MCA, basilar artery
Brekenfeld et al, 2018^12^	Germany	By ground	Retrospective cohort study	Unmatched	Driving distance between the CSC and the 2 PSCs is 53 km (hospital A) and 63 km (hospital B)	Helicopter or emergency ambulance	ICA, MCA, vertebrobasilar artery
Fernandez‐Ferro et al, 2022^13^	Spain	By ground	Retrospective cohort study	Unmatched	Between 30 and 40 min	Emergency ambulance	MCA, BA, PCA
Urbanek et al, 2023^14^	Germany	By ground	Retrospective cohort study	Unmatched	Location within 25 km or transport time of 30 min from CSC	Emergency ambulance	ICA, MCA, BA

ACA indicates anterior cerebral artery; BA, basilar artery; CSC, comprehensive stroke center; ICA, internal carotid artery; MCA, middle cerebral artery; PSC, primary stroke center; and VA, vertebral artery.

### Study Quality

The median quality grade was 9, with 6 of 11 studies having a quality grade of ≥8 for risk of bias using the NOS scoring system^3^, ^5^, ^6^, ^8^, ^9^, ^10^ (Tables S2 and S3).

### Effect of Physician Transfer on Time Interval Between Symptom Onset and Recanalization

**Figure 2:**
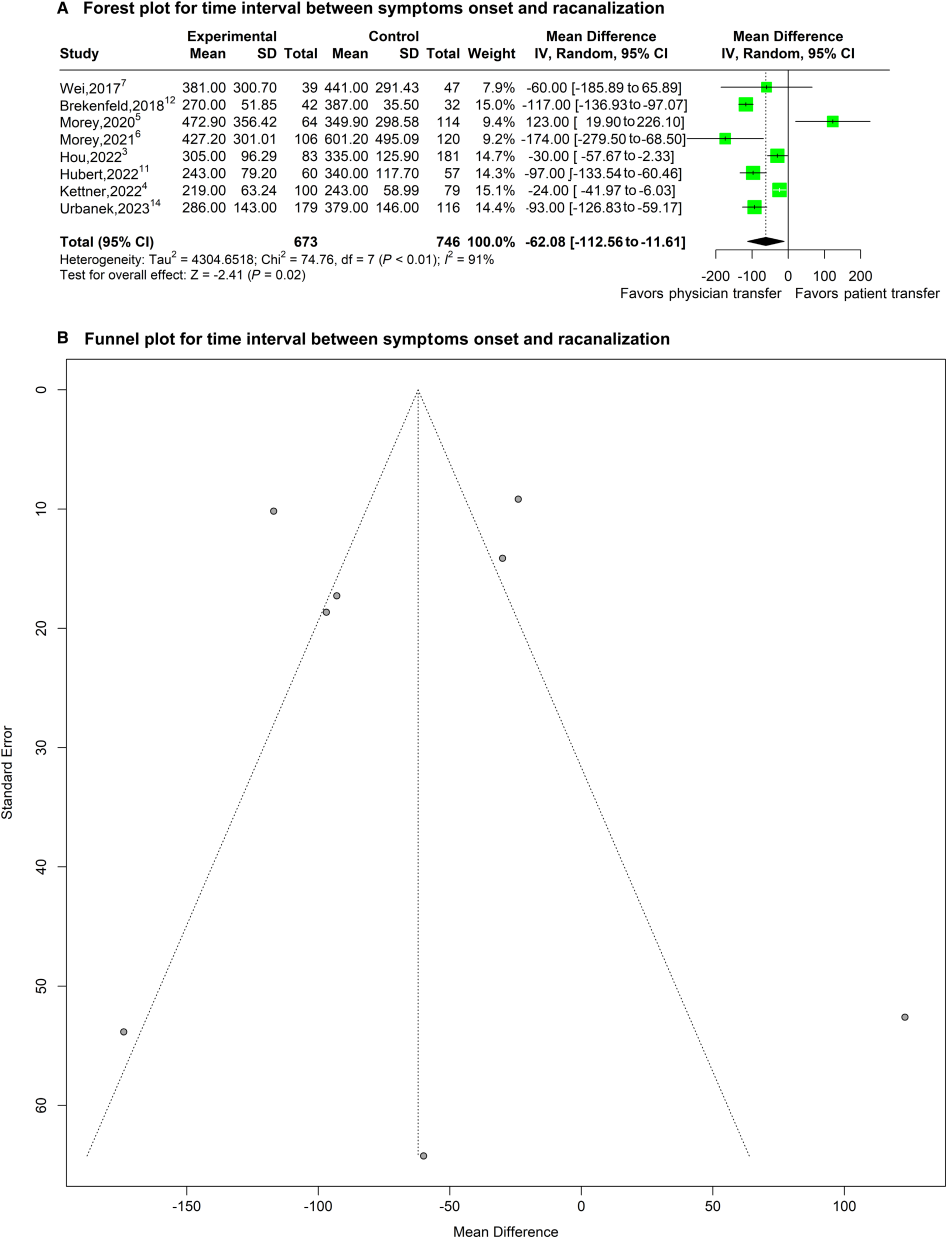
Plots of odds ratio for effect of physician transfer versus patient transfer on time interval between symptom onset and recanalization. **A**, Forest plot and hypothesis testing for heterogeneity, overall effect, and subgroup differences. **B,** Funnel plot for adjusting publication bias with published studies. IV indicates inverse variance test.

The overall mean±SD for time interval between symptom onset and recanalization was 264.6±385.7 and 340.3±409.5 minutes for physician transfer and patient transfer, respectively. Physician transfer was associated with a significantly shorter time interval between stroke onset and recanalization with a pooled mean difference estimate of −62.08 (95% CI, −112.56 to −11.61]; *P*=0.016; 8 studies involving 1419 patients [see Figure 2A]) and with high between‐study heterogeneity in the estimates (*I* ^2^=90.6%); the among‐study variance (τ^2^) is 4304.65. A sizable asymmetry was not detected in the funnel plot. After applying the trim‐and‐fill approach, the statistical significance of the resulting adjusted effect size for time interval between stroke onset and recanalization did not change, suggesting that selection bias was unlikely (Figure 2B). In the subgroup meta‐analysis stratified by the study characteristics, none of the 5 considered characteristics (country of origin, mode of physician transfer, design of trial, comparison type, and quality grade) showed any statistically significant effects on the pooled mean difference estimate (uncorrected *P* values: *P*=0.14, *P*=0.95, *P*=0.61, *P*=0.19, and *P*=0.16, respectively). The pooled mean difference estimate for time interval from stroke onset to recanalization was −26.84 (95% CI, −187.46 to 133.78]; *P*=0.74; 3 studies involving 668 patients) among studies with high‐quality score. The pooled mean difference estimate for time interval from stroke onset to recanalization was −65.25 (95% CI, −106.88 to −23.62]; *P*=0.02; 5 studies involving 898 patients) among studies that used unmatched comparators. The overall median±interquartile range for the time interval between symptom onset and recanalization was 295.5 (263.25–392.55) minutes and 364.45 (338.75–400.50) minutes for physician transfer and patient transfer, respectively. Physician transfer significantly reduced the median time interval between stroke onset and recanalization with a pooled median difference estimate of −62.67 (95% CI, −100.81 to −24.52]; *P*=0.001; 8 studies involving 1419 patients [see Figure S1]) and with high between‐study heterogeneity in the estimates (*I* ^2^=86.6%), and the among‐study variance (τ^2^) is 2402.87.

### Effect of Physician Transfer on Functional Independence at 90 Days

**Figure 3:**
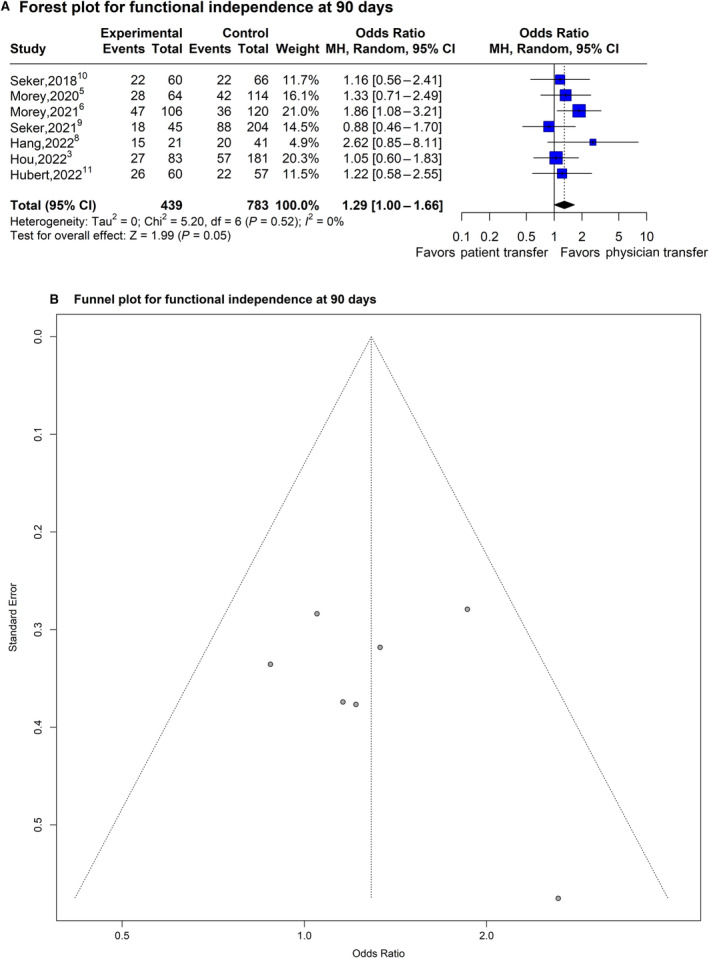
Plots of odds ratio for effect of physician transfer versus patient transfer on functional independence at 90 days (modified Rankin Scale score, 0–2). **A**, Forest plot and hypothesis testing for heterogeneity, overall effect, and subgroup differences. **B**, Funnel plot for adjusting publication bias with published studies. MH indicates, Mantel–Haenszel test.

The OR for functional independence at 90 days was significantly higher (OR, 1.29 [95% CI, 1.00–1.66]; *P*=0.046; 7 studies involving 1222 patients) with physician transfer with low between‐study heterogeneity (*I* ^2^=0%) (Figure 3A). A sizable asymmetry was not detected in the funnel plot. After applying the trim‐and‐fill approach, the statistical significance of the resulting adjusted OR for functional independence at 90 days did not change, suggesting that selection bias was unlikely (Figure 3B). Similar results were observed in a fixed‐effect model (OR, 1.29 [95% CI, 1.00–1.66]; *P*=0.42). In the subgroup meta‐analysis stratified by the study characteristics, 4 of the 5 considered characteristics (country of origin, mode of physician transfer, design of trial, and comparison type) did not show any statistically significant effects on the OR estimate for functional independence at 90 days (uncorrected *P* values: *P*=0.36, *P*=0.87, *P*=0.95, and *P*=0.18, respectively). All studies included had the same quality grade, so further stratification was not possible.

### Effect of Physician Transfer on All‐Cause Death Within 90 Days

**Figure 4:**
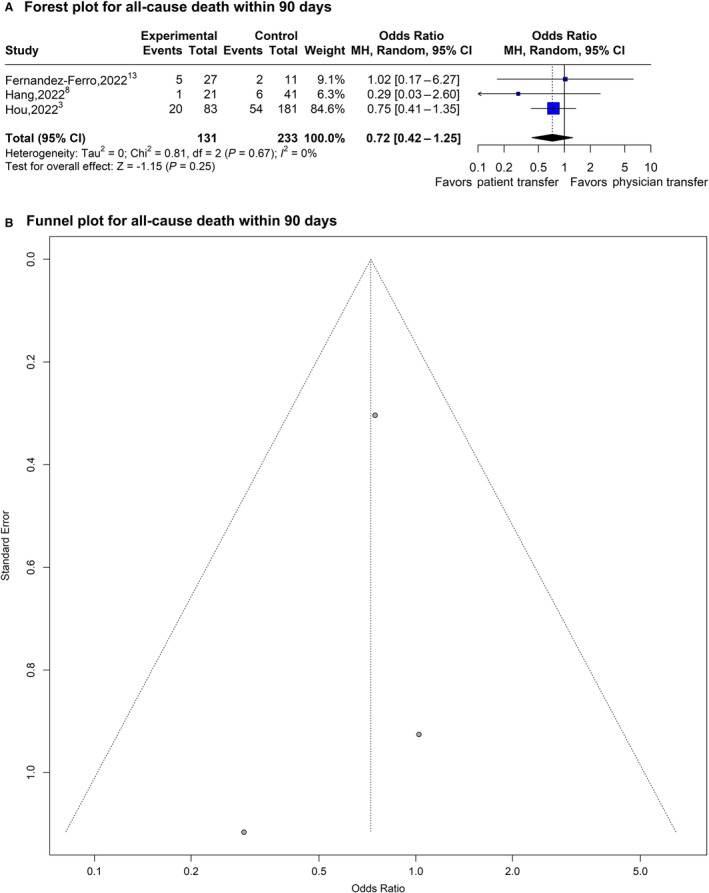
Plots of odds ratio for effect of physician transfer versus patient transfer on all‐cause death within 90 days. **A**, Forest plot and hypothesis testing for heterogeneity, overall effect, and subgroup differences. **B,** Funnel plot for adjusting publication bias with published studies. MH indicates, Mantel–Haenszel test.

The OR for all‐cause death within 90 days with physician transfer was 0.72 (95% CI, 0.42–1.25; *P*=0.25; *I* ^2^=0%; 3 studies, 364 subjects; Figure 4A). A sizable asymmetry was not detected in the funnel plot. After applying the trim‐and‐fill approach, the statistical significance of the resulting adjusted OR for all‐cause death at 90 days did not change, suggesting that selection bias was unlikely (Figure 4B). Similar results were observed in a fixed‐effect model (OR, 0.71 [95% CI, 0.41–1.22]; *P*=0.22). In the subgroup meta‐analysis stratified by the study characteristics (country of origin, mode of physician transfer, design of trial, comparison type, and quality grade), neither country of origin nor quality grade showed any statistically significant effects on the OR estimate for all‐cause death within 90 days (uncorrected *P* values: *P*=0.70 and *P*=0.70, respectively), and all 3 studies had the same mode of physician transfer, design of trial, and a matching comparison for both cases and controls, so further stratification was not possible. The sensitivity analysis using leave‐one‐out meta‐analysis provided an OR of 0.71 (95% CI, 0.41–1.22; *P*=0.22).

### Effect of Physician Transfer on Post‐MT sICH

**Figure 5:**
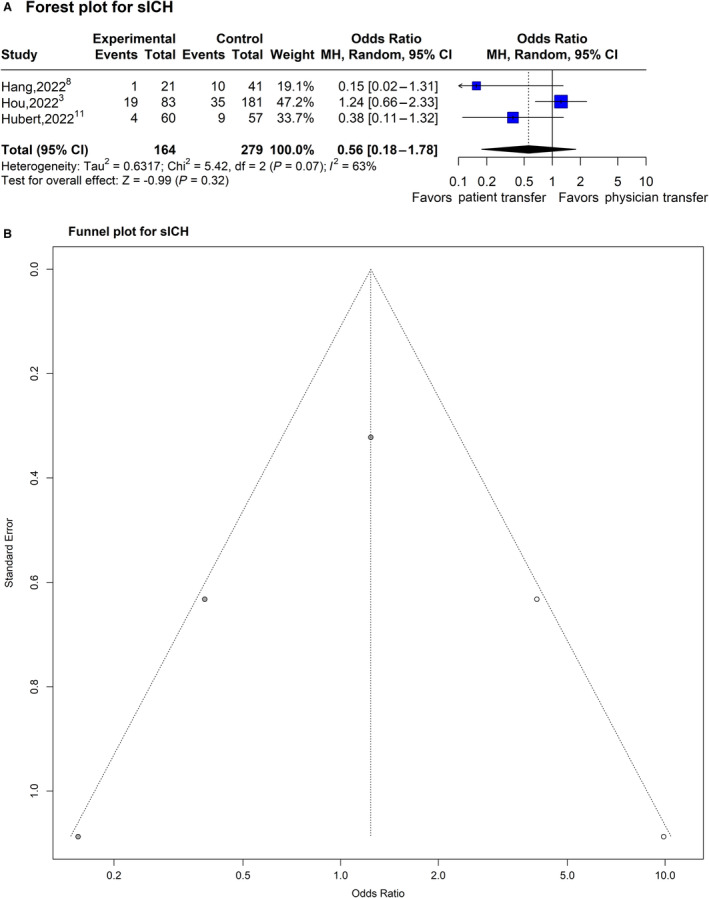
Plots of odds ratio for effect of physician transfer versus patient transfer on postthrombectomy symptomatic intracranial hemorrhage. **A**, Forest plot and hypothesis testing for heterogeneity, overall effect, and subgroup differences. **B,** Funnel plot for adjusting publication bias with published studies (filled circles) and trim‐and‐fill assigned studies (open circles). MH indicates Mantel–Haenszel test; and sICH, symptomatic intracerebral hemorrhage.

The OR for post‐MT sICH with physician transfer was 0.56 (95% CI, 0.18–1.78; *P*=0.32; *I* ^2^=63.1%; 3 studies, 443 subjects; Figure 5A). A sizable asymmetry was detected in the funnel plot, suggesting the presence of a publication bias. After applying the trim‐and‐fill approach, the 2 missing experiments were imputed, and the adjusted OR for sICH was 1.24 (95% CI, 0.34–4.47; *P*=0.74; Figure 5B). In the subgroup meta‐analysis stratified by the study characteristics, none of the 5 considered characteristics (country of origin, mode of physician transfer, design of trial, comparison type, and quality grade) showed any statistically significant effects on the OR estimate for post‐MT sICH (uncorrected *P* values: *P*=0.74, *P*=0.74, and *P*=0.74, respectively). All 3 studies had the same matching comparison for both cases and controls and quality grade, so further stratification was not possible. The sensitivity analysis using leave‐one‐out meta‐analysis provided an OR of 0.79 (95% CI, 0.47–1.34; *P*=0.38).

### Effect of Physician Transfer on Near‐Complete or Complete Angiographic Recanalization

**Figure 6:**
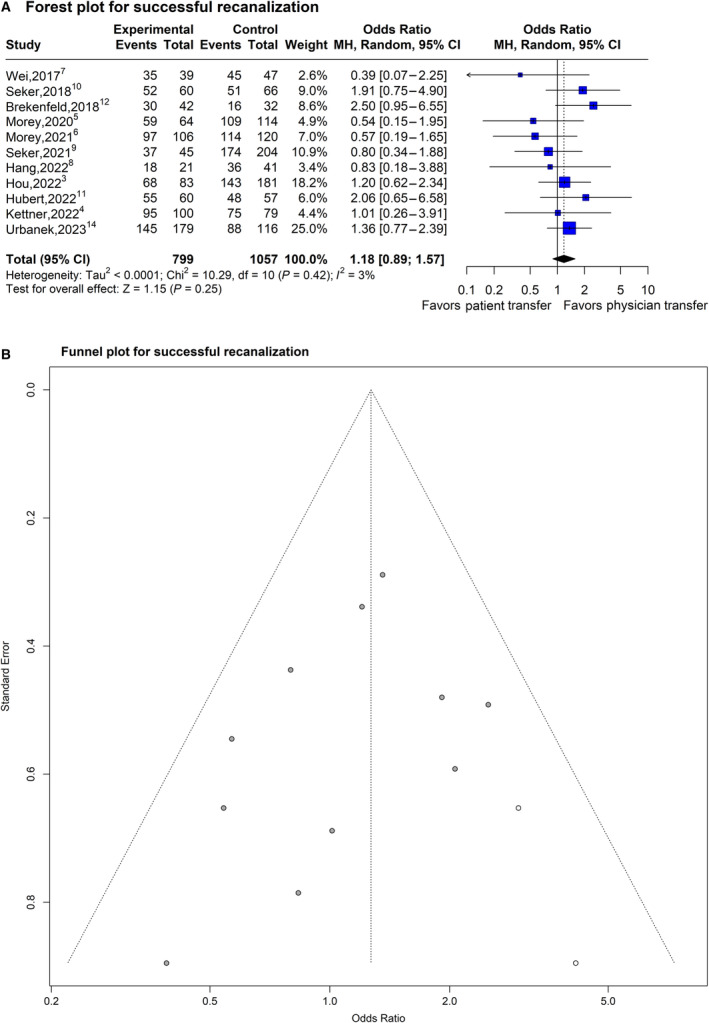
Plots of odds ratio for effect of physician transfer versus patient transfer on near‐complete or complete angiographic recanalization after mechanical thrombectomy. **A**, Forest plot and hypothesis testing for heterogeneity, overall effect, and subgroup differences. **B,** Funnel plot for adjusting publication bias with published studies (filled circles) and trim‐and‐fill assigned studies (open circles). MH indicates Mantel–Haenszel test.

The OR for successful recanalization with physician transfer was 1.18 (95% CI, 0.89–1.57; *P*=0.25; *I* ^2^=2.8%; 11 studies, 1856 subjects; Figure 6A). A sizable asymmetry was detected in the funnel plot. After applying the trim‐and‐fill approach, the 2 missing experiments were imputed, and the adjusted OR for near‐complete or complete angiographic recanalization was higher with physician transfer (OR, 1.27 [95% CI, 0.97–1.67]; *P*=0.09; Figure 6B). In the subgroup meta‐analysis stratified by the study characteristics, none of the 5 considered features (country of origin, mode of physician transfer, design of trial, comparison type, and quality grade) showed any statistically significant effects on the OR estimate for near complete or complete angiographic recanalization (Bonferroni adjusted *P*=0.09, *P*=0.55, *P*=0.08, *P*=0.07, and *P*=0.26, respectively).

## Discussion

### Salient Findings

We found that there was a significant reduction in mean time interval between symptom onset and recanalization in patients who were treated with physician transfer compared with those treated with patient transfer (overall mean±SD for time interval 264.6 and 340.3 minutes; average difference, 75.7 minutes). Previous studies have identified a strong relationship between time interval from symptom onset to recanalization and odds of functional independence.^26^, ^27^, ^28^, ^29^, ^30^ He et al^26^ identified that every 15‐minute delay in time interval between symptom onset and recanalization, the odds of functional independence decreased by 10%. Khatri et al^27^ reported that time interval between symptom onset and recanalization of 310 minutes, compared with 280 minutes, corresponded to a 10.6% decrease in the probability of functional independence. Menon et al^29^ identified that each 1‐hour increase in time interval between symptom onset and recanalization reduced the odds of functional independence by 38%. The probability of functional independence declined from 64.1% to 46.1% with increase in time interval between symptom onset and recanalization from 180 to 480 minutes. An analysis of the ESCAPE (Endovascular Treatment for Small Core and Proximal Occlusion Ischemic Stroke) trial found that every 30‐minute increase in time from stroke symptom onset to reperfusion was associated with an absolute reduction in the probability of functional independence by 1.9%.^31^ In our meta‐analysis, the odds for functional independence at 90 days was significantly higher with physician transfer (compared with patient transfer) with low between‐study heterogeneity. The higher odds are probably related to significant reduction in mean time interval between symptom onset and recanalization among patients treated with physician transfer as discussed earlier. However, part of the higher rates of functional independence may be related to the higher odds for successful recanalization with physician transfer in our analysis. There was no difference in the odds of post‐MT sICH between patients treated with physician transfer compared with those treated with patient transfer.

### Generalizability and Implementation

The superiority of MT using physician transfer (compared with patient transfer) raises the question of both generalizability and potential for implementation. Our literature search started from January 2012, but all studies included were from 2017 onward. Therefore, we think that data are representative of the current stroke systems of care including various recent American Heart Association/American Stroke Association initiatives such as establishment of stroke systems of care.^32^

Hubert et al^11^ compared the results of physician transfer and patient transfer (alternating weeks) in a network of 13 primary stroke centers of the telestroke network TEMPiS located in rural or intermediate populated areas in Germany, which covered a region of 11 295 km^2^ with a population of 1.54 million. The network treated ≈5500 patients with acute stroke per year. All primary stroke centers had a neurology department or internal medicine department with a stroke unit, an intensive care unit, a helicopter pad, a monoplane angiography suite, and onsite anesthesia support, and provided further support by an angiography assistant. Five CSCs in the wider region of the network serve as MT referral centers for these hospitals. A helicopter service was dedicated exclusively for the physician transfer. A total of 72 patients were treated with physician transfer, and 85 patients were treated by patient transfer. The median time from the decision to pursue MT to the start of the MT was 58 minutes in patients treated by physician transfer and 148 minutes in those who were treated by patient transfer. The significant reduction in time interval between the decision to pursue MT to the start of the MT resulted in premature completion of the study.^11^ The study by Hubert et al^11^ outlines some of the prerequisites for implementation of physician transfer such as the satellite site to have additional capabilities compared with hospitals that only receive and transfer the patients with acute ischemic stroke. The satellite site must have an angiographic unit capable of performing MT, nurses and technicians, and necessary devices such as catheters and MT devices.^33^ These capabilities must be available throughout the 24‐hour period with a rapid triage and transfer to the angiographic suite. Hospitals that provide percutaneous coronary intervention and do not provide MT may serve as satellite sites for physician transfer. There are ≈1700 hospitals in the United States that provide percutaneous coronary intervention.^34^, ^35^ Some data show that MT can be performed in interventional cardiology suites in a time‐efficient manner.^36^, ^37^, ^38^ However, additional issues such as hospital privileges and working between different hospital systems need to be considered. Additionally, interventional cardiology suites lack nurses and technicians with specific experience in neurointerventional procedures and equipment (aspiration catheters and stent retrievers) required for MT. The issue was addressed in the study by Hubert et al^11^ by adding a technician nurse as part of the flying neurointerventional team and catheters and other equipment required for MT were brought by the team. Another aspect that needs to be considered before implementation is the different costs incurred between patient transfer and physician transfer paradigms for providing MT to patients with acute ischemic stroke.^39^

In the study by Hubert et al,^11^ the decision to pursue MT was identical in both groups and consisted of a combination of radiographic confirmation and clinical examination. The significant time saving in these models came from parallelization of processes. During the transfer time in physician transfer, the patients were already being prepared in the local angiographic suite. In patient transfer, the patients cannot be prepared until arrival to the CSC angiographic suite following transfer from initial hospital. The transfer time is also shorter for transferring physicians (and neurointerventional team) compared with a patient with ischemic stroke who requires monitoring, intravenous infusions, and cardiorespiratory support. The possibility of transferring the neurointerventional team to the initial hospital for a potential patient undergoing MT who may not eventually require MT should be considered. In the study by Hubert et al,^11^ MT was not performed after physician transfer or patient transfer because of clinical improvement or spontaneous recanalization (13% and 16%). Interestingly, an additional 14% of the patients in the patient transfer group did not receive MT due to lack of salvageable brain tissue or technical challenges. Only an additional 4% of the patients in the physician transfer did not receive MT due to technical challenges.

Another potential benefit of physician transfer is to create a more favorable ratio between the number of neurointerventionalists and procedural volume for each neurointerventionalist. Currently, hospitals with small procedural volume of MT may not transfer patients to CSCs and hire their own neurointerventionalist as evident by over a quarter of MTs performed at hospitals without advanced capability accreditation.^40^ Such an arrangement requires a much larger number of neurointerventionalists but with low procedural volume. An analysis of national inpatient Medicare data reported that 13 335 patients aged ≥65 years were treated by 2754 interventionalists at 641 hospitals from January 1, 2016, to December 31, 2017.^41^ There was considerable variation in procedural volume among interventionalists with reduction in inpatient death (4% lower odds for every 10 additional MTs performed per year) and increase in discharge home or inpatient rehabilitation (3% greater adjusted odds for every 10 additional MTs performed per year). Physician transfer will allow a smaller number of neurointerventionalists to provide MT at multiple hospitals while maintaining procedural volumes that ensure favorable outcomes.

Other issues, such as the disadvantage of taking a neurointerventionalist out of coverage at a CSC to transport them to a smaller hospital to perform MT and additionally an emergency medical services unit or helicopter out of prehospital patient transport duty to transfer the physician, need to be considered. In the event that a neurointerventionalist is performing MT at the initial hospital after physician transfer, the availability of a neurointerventionalist at the CSC in the event of concurrent MT may be compromised. However, the gap can be addressed with the availability of backup neurointerventionalists at CSCs. It should be noted that even if the patient at the initial hospital was transferred to a CSC, the neurointerventionalist and angiographic suite may still be unavailable due to the ongoing MT. In that regard, physician transfer may allow use of the angiographic unit and staff at the initial hospital as long as a backup neurointerventionalist is available at the CSC. In the absence of backup neurointerventionalist at the CSC, back‐to‐back availability of a neurointerventionalist is hindered by the transfer time for the neurointerventionalist between hospitals, which may not be a concern if both procedures are performed at the CSC.

Although the number of health care systems that can adopt a physician transfer paradigm is not known, a nonstandardized survey of participants at Health Services, Quality Improvement, and Patient Centered Outcomes session of the International Stroke Conference (February 9, 2024), 64% of the attendees demonstrated willingness to implement a physician transfer protocol in their current practice.

### Limitations

The analysis is predominantly based on observational studies. Although observational studies are included in 64% of systematic reviews,^42^, ^43^, ^44^ we acknowledge the limitations posed by observational studies in determining the direct effect of physician transfer on various outcomes due to both bias and confounding.^45^ There was both qualitative and quantitative evidence of heterogeneity between studies. There was high between‐study heterogeneity in the estimate of the effect of physician transfer on the time interval between symptom onset and recanalization (*I* ^2^=91.7%). Large heterogeneity may lead to potentially misleading results.^46^ We used a random‐effects model and performed subgroup analysis and meta‐regression using study‐level characteristics as recommended by Cordero and Dans.^46^ We also performed several sensitivity analyses to confirm the results. We were unable to perform analysis of individual‐level data, which may provide additional insight into the observed heterogeneity.

The results are derived from selected centers and multiple countries, and physician transfer is based predominantly on ground transport. Apart from methodological heterogeneity, clinical heterogeneity is also expected to be much higher since our analysis includes mainly observational studies that are based on less stringent inclusion criteria.^43^ Another challenge in meta‐analyses of observational studies is pooling the adjusted results.^47^ We used crude unadjusted numbers to avoid the biases introduced by different methods of adjustment within each study. We used the NOS for cohort studies, which is a commonly used method of grading the quality of observational studies.^48^, ^49^ The median quality grade of the studies was 9. Six of 11 studies had a quality grade of ≥8 for risk of bias using the NOS scoring system. We performed a sensitivity analysis excluding studies of lower‐quality grade as recommended by Metelli and Chaimani.^45^ We also noticed funnel plot asymmetry in all the end points ascertained, which can be a result of publication or other reporting biases, heterogeneity, and chance.^50^, ^51^ We used the random‐effects model to account for heterogeneity and performed subgroup analysis or meta‐regression by study design and characteristics, as recommended by Metelli and Chaimani.^45^ However, due to the small number of studies, our meta‐regression was limited since a large ratio of studies to a covariate is required for a meaningful analysis. Usually, a ratio of 10 studies to 1 covariate is recommended in meta‐regression.^52^ Our analysis was not able to identify the proportion of patients who were excluded from receiving MT in the patient transfer group due to excessive delay incurred during transfer.

The issue of small number of events in the meta‐analysis was evident when considering the analyses of death and sICH. Hang et al.^8^ report only 1 event in the physician transfer group for both death and sICH outcomes. In our analyses, the study by Hang et al contributed limited weight to both death and sICH analyses, effectively minimizing the consequences resulting from rare events in the analyses. Notably, there are no studies in our analysis with 0 events, and therefore, we did not apply the method described by Efthimiou,^53^ which primarily addresses challenges posed by single‐0 or double‐0 studies, signifying 0 events in experimental/control arms. We also performed a sensitivity analysis using a leave‐one‐out meta‐analysis to limit the exaggerated effect of some studies.^21^

## Conclusions

The significant reduction in the mean time interval between symptom onset and recanalization associated with physician transfer (compared with patient transfer) among patients treated with MT supports further exploration of this strategy to optimize the results of MT in patients with acute ischemic stroke. Physician transfer also has the potential to increase the delivery of MT to rural populations in the United States, which are less likely to receive MT.^54^ Further research may require studies using cluster randomization designs using comparable health systems, with one health system adopting a physician transfer strategy and another using a patient transfer strategy or alternating time periods such as used by Hubert et al^11^ due to challenges posed by standard randomized designs.^55^

## Sources of Funding

None.

## Disclosures

None.

## Supporting information

Data S1
Supplemental Methods
Tables S1–S3
Figure S1
