# Supplementary material for: Physician Transfer Versus Patient Transfer for Mechanical Thrombectomy in Patients With Acute Ischemic Stroke: A Systematic Review and Meta‐Analysis
Source: J Am Heart Assoc. 2024 Jun 20;13(13):e031906. doi: 10.1161/JAHA.123.031906 (PMC11255715; doi:10.1161/JAHA.123.031906)

Supplemental Material

Data S1. Supplemental Methods

Reporting checklist for systematic review (with or without a meta-analysis)

Based on the PRISMA guidelines.

		Reporting Item	Page Number
**Title**			
Title	#1	Identify the report as a systematic review	1
**Abstract**			
Abstract	#2	Report an abstract addressing each item in the PRISMA 2020 for Abstracts checklist	2, 3
**Introduction**			
Background/rationale	#3	Describe the rationale for the review in the context of existing knowledge	5
Objectives	#4	Provide an explicit statement of the objective(s) or question(s) the review addresses	5
**Methods**			
Eligibility criteria	#5	Specify the inclusion and exclusion criteria for the review and how studies were grouped for the syntheses	6
Information sources	#6	Specify all databases, registers, websites, organisations, reference lists, and other sources searched or consulted to identify studies. Specify the date when each source was last searched or consulted	6
Search strategy	#7	Present the full search strategies for all databases, registers, and websites, including any filters and limits used	5, 6
Selection process	#8	Specify the methods used to decide whether a study met the inclusion criteria of the review, including how many reviewers screened each record and each report retrieved, whether they worked independently, and, if applicable, details of automation tools used in the process	6, 7
Data collection process	#9	Specify the methods used to collect data from reports, including how many reviewers collected data from each report, whether they worked independently, any processes for obtaining or confirming data from study investigators, and, if	7

		applicable, details of automation tools used in the process	
Data items	#10a	List and define all outcomes for which data were sought. Specify whether all results that were compatible with each outcome domain in each study were sought (for example, for all measures, time points, analyses), and, if not, the methods used to decide which results to collect	7
Study risk of bias assessment	#11	Specify the methods used to assess risk of bias in the included studies, including details of the tool(s) used, how many reviewers assessed each study and whether they worked independently, and, if applicable, details of automation tools used in the process	7, 8
Effect measures	#12	Specify for each outcome the effect measure(s) (such as risk ratio, mean difference) used in the synthesis or presentation of results	8
Synthesis methods	#13a	Describe the processes used to decide which studies were eligible for each synthesis (such as tabulating the study intervention characteristics and comparing against the planned groups for each synthesis (item #5))	7
Synthesis methods	#13b	Describe any methods required to prepare the data for presentation or synthesis, such as handling of missing summary statistics or data conversions	7
Synthesis methods	#13c	Describe any methods used to tabulate or visually display results of individual studies and syntheses	7
Synthesis methods	#13d	Describe any methods used to synthesise results and provide a rationale for the choice(s). If meta-analysis was performed, describe the model(s), method(s) to identify the presence and extent of statistical heterogeneity, and software package(s) used	8, 9
Synthesis methods	#13e	Describe any methods used to explore possible causes of heterogeneity among study results (such as subgroup analysis, meta-regression)	8
Synthesis methods	#13f	Describe any sensitivity analyses conducted to assess robustness of the synthesised results	8
Reporting bias assessment	#14	Describe any methods used to assess risk of bias due to missing results in a synthesis (arising from reporting biases)	8

Certainty assessment	#15	Describe any methods used to assess certainty (or confidence) in the body of evidence for an outcome	8
Data items	#10b	List and define all other variables for which data were sought (such as participant and intervention characteristics, funding sources). Describe any assumptions made about any missing or unclear information	7
**Results**			
Study selection	#16a	Describe the results of the search and selection process, from the number of records identified in the search to the number of studies included in the review, ideally using a flow diagram (http://www.prisma-statement.org/PRISMAStatement/FlowDiagram)	9
Study selection	#16b	Cite studies that might appear to meet the inclusion criteria, but which were excluded, and explain why they were excluded	9
Study characteristics	#17	Cite each included study and present its characteristics	9, 28
Risk of bias in studies	#18	Present assessments of risk of bias for each included study	9
Results of individual studies	#19	For all outcomes, present for each study (a) summary statistics for each group (where appropriate) and (b) an effect estimate and its precision (such as confidence/credible interval), ideally using structured tables or plots	10-12
Results of syntheses	#20a	For each synthesis, briefly summarise the characteristics and risk of bias among contributing studies	9
Results of syntheses	#20b	Present results of all statistical syntheses conducted. If meta-analysis was done, present for each the summary estimate and its precision (such as confidence/credible interval) and measures of statistical heterogeneity. If comparing groups, describe the direction of the effect	9-12
Results of syntheses	#20c	Present results of all investigations of possible causes of heterogeneity among study results	9-12
Results of syntheses	#20d	Present results of all sensitivity analyses conducted to assess the robustness of the synthesised results	9-12

Risk of reporting biases in syntheses	#21	Present assessments of risk of bias due to missing results (arising from reporting biases) for each synthesis assessed	9-12
Certainty of evidence	#22	Present assessments of certainty (or confidence) in the body of evidence for each outcome assessed	9-12
**Discussion**			
Results in context	#23a	Provide a general interpretation of the results in the context of other evidence	13, 14
Limitations of included studies	#23b	Discuss any limitations of the evidence included in the review	17, 18
Limitations of the review methods	#23c	Discuss any limitations of the review processes used	17, 18
Implications	#23d	Discuss implications of the results for practice, policy, and future research	14-17
**Other information**			
Registration and protocol	#24a	Provide registration information for the review, including register name and registration number, or state that the review was not registered	5
Registration and protocol	#24b	Indicate where the review protocol can be accessed, or state that a protocol was not prepared	5
Registration and protocol	#24c	Describe and explain any amendments to information provided at registration or in the protocol	5
Support	#25	Describe sources of financial or non-financial support for the review, and the role of the funders or sponsors in the review	19
Competing interests	#26	Declare any competing interests of review authors	19
Availability of data, code, and other materials	#27	Report which of the following are publicly available and where they can be found: template data collection forms; data extracted from included studies; data used for all analyses; analytic code; any other materials used in the review	19

Search Syntax

Search syntax used in Medline:

(("patient transfer" or "Time Factors" or "emergency medical services" or "mobile health units" or "transport strategy" or "drive doctor" or "drip ship" or "drip drive" or "flying intervention team" or "interhospital transfer" or "triage") and ("mechanical thrombectomy" or "Thrombectomy" or "endovascular procedure" or "intravascular procedure" or "embolectomy" or "endovascular" or "mechanical clot disruption" or "mechanical thrombolysis" or "transcatheter") and ("Acute ischemic stroke" or "ischemic stroke" or "stroke" or "strokes" or "large vessel occlusion" or "brain ischemia" or "brain infarction" or "cerebral infarction" or "cerebral ischemia" or "middle cerebral artery" or "MCA" or "anterior circulation" or "posterior circulation" or "cerebrovascular disease" or "cerebrovascular accident" or "transcatheter")).ab.

Search syntax used in the database of web of science to search studies after 2011:

(((TS=("Acute ischemic stroke" OR "ischemic stroke" OR stroke OR strokes OR "large vessel occlusion" OR "brain ischemia" OR "brain infarction" OR "cerebral infarction" OR "cerebral ischemia" OR "middle cerebral artery" OR MCA OR "anterior circulation" OR "posterior circulation" OR "cerebrovascular disease" OR "cerebrovascular accident")) AND TS=("Mechanical thrombectomy" OR thrombectomy OR "endovascular procedure" OR "intravascular procedure" OR embolectomy OR endovascular OR "mechanical clot disruption" OR "mechanical thrombolysis" OR transcatheter))) AND TS=("patient transfer" OR "Time Factors" OR "emergency medical services" OR "mobile health units" OR

"transport strategy" OR "drive doctor" OR "drip ship" OR "drip drive" OR "flying intervention team" OR "interhospital transfer" OR "triage")

Search syntax used in the database of Scopus to search studies after 2011:

(TITLE-ABS-KEY ("Acute ischemic stroke" OR "ischemic stroke" OR stroke OR strokes OR "large vessel occlusion" OR "brain ischemia" OR "brain infarction" OR "cerebral infarction" OR "cerebral ischemia" OR "middle cerebral artery" OR mca OR "anterior circulation" OR "posterior circulation" OR "cerebrovascular disease" OR "cerebrovascular accident") AND TITLE-ABS-KEY ("Mechanical thrombectomy" OR thrombectomy OR "endovascular procedure" OR "intravascular procedure" OR embolectomy OR endovascular OR "mechanical clot disruption" OR "mechanical thrombolysis" OR transcatheter) AND TITLE-ABS-KEY ("observational study" OR "clinical trial" OR "Clinical study" OR "Clinical trials" OR "Comparative studies" OR "Controlled clinical trials" OR "Multicenter studies" OR "Observational studies" OR "Randomized controlled trials") AND TITLE-ABS-KEY ("patient transfer" OR "Time Factors" OR "emergency medical services" OR "mobile health units" OR "transport strategy" OR "drive doctor" OR "drip ship" OR "drip drive" OR "flying intervention team" OR "interhospital transfer" OR "triage"))

Search syntax used in Pubmed to search studies after 2011 and we applied further filters of clinical study, clinical trial, multicenter study, observational study, randomized controlled trial, comparative studies:

((("patient transfer"[MeSH] OR "Time Factors"[MeSH] OR "emergency medical services"[MeSH] OR "mobile health units"[MeSH] OR transport strategy OR drive doctor OR drip ship OR drip drive OR flying intervention team OR interhospital transfer OR triage)) AND (("mechanical thrombectomy"[Title/Abstract] OR "Thrombectomy"[Title/Abstract] OR "endovascular procedure"[Title/Abstract] OR "intravascular procedure"[Title/Abstract] OR "embolectomy"[Title/Abstract] OR "endovascular"[Title/Abstract] OR "mechanical clot disruption"[Title/Abstract] OR "mechanical thrombolysis"[Title/Abstract] OR "transcatheter"[All Fields] OR "Thrombectomy"[MeSH Terms] OR "Thrombectomy"[MeSH Terms]))) AND ((Acute ischemic stroke[Title/Abstract] OR ischemic stroke[Title/Abstract] OR stroke[Title/Abstract] OR strokes[Title/Abstract] OR large vessel occlusion[Title/Abstract] OR brain ischemia[Title/Abstract] OR brain infarction[Title/Abstract] OR cerebral infarction[Title/Abstract] OR cerebral ischemia[Title/Abstract] OR middle cerebral artery[Title/Abstract] OR MCA[Title/Abstract] OR anterior circulation[Title/Abstract] OR posterior circulation[Title/Abstract] OR cerebrovascular disease[Title/Abstract] OR cerebrovascular accident[Title/Abstract] OR transcatheter[Title/Abstract] OR "Stroke"[Mesh])) AND ((clinicaltrial[Filter] OR comparativestudy[Filter] OR controlledclinicaltrial[Filter] OR multicenterstudy[Filter] OR observationalstudy[Filter]) AND (2012:2023[pdat]))

Table S1. Definitions of symptomatic intracranial hemorrhage and successful reperfusion in included studies.

Author	Successful reperfusion	Symptomatic intracranial hemorrhage
Brekenfeld, C., et al. 2018[12]	TICI 2b/3	NA
Fernandez-Ferro, J., et al. 2022[13]	NA	NA
Hang, Y., et al. 2022[8]	mTICI 2b/3	sICH definition not available
Hou, J., et al. 2022[3]	Vascular recanalization was defined as modified Thrombolysis in Cerebral Infarction Score (mTICI) 2B-3	sICH was defined as any type of intracranial hemorrhage with an increase in NIHSS of greater than or equal to 4 points or death within 24 hours
Hubert, G.J., et al. 2022[11]	mTICI ≥2b	Any postprocedural intracranial hemorrhage and worsening of the neurological deficit of greater than or equal to 4 points on the National Institute of Health Stroke Scale, irrespective of causality or location.

Kettner, A., et al. 2022 [4]	mTICI ≥2b	NA
Morey, J.R., et al. 2020 [5]	mTICI ≥2b	NA
Morey, J.R., et al. 2021 [6]	TICI ≥2b	NA
Seker, F., et al. 2021 [9]	TICI 2b/3	NA
Seker, F., et al. 2018 [10]	TICI 2b-3	NA
Urbanek, C., et al. 2023 [14]	TICI 2b-3	NA
Wei, D., et al. 2017 [7]	TICI 2b-3	NA

sICH = symptomatic intracranial hemorrhage, NIHSS = National Institutes of Health Stroke Scale/Score, TICI, Thrombolysis in Cerebral Infarction, mTICI, modified Thrombolysis in Cerebral Infarction, NA = not applicable

Table S2. Quality score of included observational studies.

Study	Selection				Comparability	Outcome			Final score
	Representativeness of the exposed cohort	Selection of the non-exposed cohort	Ascertainment of exposure	Demonstration that outcome of interest was not present at start of study	Comparability of cohorts on the basis of the design or analysis controlled for confounders	Assessment of outcome	Was follow-up long enough for outcomes to occur	Adequacy of follow-up of cohorts	
Brekenfeld, C., et al. 2018[12]	*	*	*	*	** Demographic data and patient characteristics were similar	*	No star no statement	No star no statement	7

Fernandez-Ferro, J., et al. 2022[13]	*	*	*	*	** Baseline clinical, stroke-related and radiological characteristics are comparable between groups	*	No star no statement	No star no statement	7
Hang, Y., et al. 2022[8]	*	*	*	*	** Demographic data and patient characteristics were similar	*	* (functional outcomes measured at 3 months follow-up)	*	9
Hou, J., et al. 2022[3]	*	*	*	*	** Adjusted for age and gender Controlled for potential confounders	*	* (functional outcomes measured at 3 months follow-up)	*	9

Hubert, G.J., et al. 2022[11]	NA	NA	NA	NA	NA	NA	NA	NA	NA
Kettner, A., et al. 2022[4]	*	*	*	*	** patient characteristics were similar at baseline	*	No star no statement	No star no statement	7
Morey, J.R., et al. 2020[5]	*	*	*	*	** Groups were similarly matched in terms of baseline characteristics.	*	* (functional outcomes measured at 3 months follow-up)	*	9

***Morey, J.R., et al. 2021*[6]**	*	*	*	*	** Adjusted for diabetes, hypertension, previous ischemic stroke or transient ischemic attack, and occlusion location, were used to verify robustness of the results Propensity score matching methods, adjusted for unbalanced, confounding factors, were used	*	* (functional outcomes measured at 3 months follow-up)	*	9

Seker, F., et al. 2021[9]	*	*	*	*	** Groups were similar in terms of baseline characteristics.	*	* (functional outcomes measured at 3 months follow-up)	*	9
Seker, F., et al. 2018[10]	*	*	*	*	** Groups were similar in terms of baseline characteristics.	*	* (functional outcomes measured at 3 months follow-up)	*	9
Urbanek, C., et al. 2023[14]	*	*	*	*	** Groups were similarly matched regarding baseline characteristics	*	No star no statement	No star no statement	7

Wei, D., et al. 2017[7]	*	*	*	*	** Groups were similarly matched regarding baseline characteristics	*	No star no statement	No star no statement	7

Table S3. Quality assessment by Methodological index for non-randomized study (MINORS) (Hubert, G.J., et al. 2022).[11]

A clearly stated aim	2
Inclusion of consecutive patients	2
Prospective collection of data	2
Endpoints appropriate to the aim of the study	2
Unbiased assessment of the study endpoint	0
Follow-up period appropriate to the aim of the study	2
Loss to follow up less than 5%	2
Prospective calculation of the study size	2
An adequate control group	2
Contemporary groups	0
Baseline equivalence of groups	2
Adequate statistical analyses	2

Every item use "Not reported (0 point)", "Reported but inadequate (1 point), or "Reported and adequate (2 point)" to judge.

Figure S1. Forest plot (based on median values) of odds ratio for effect of physician transfer versus patient transfer on time interval between symptom onset and recanalization.

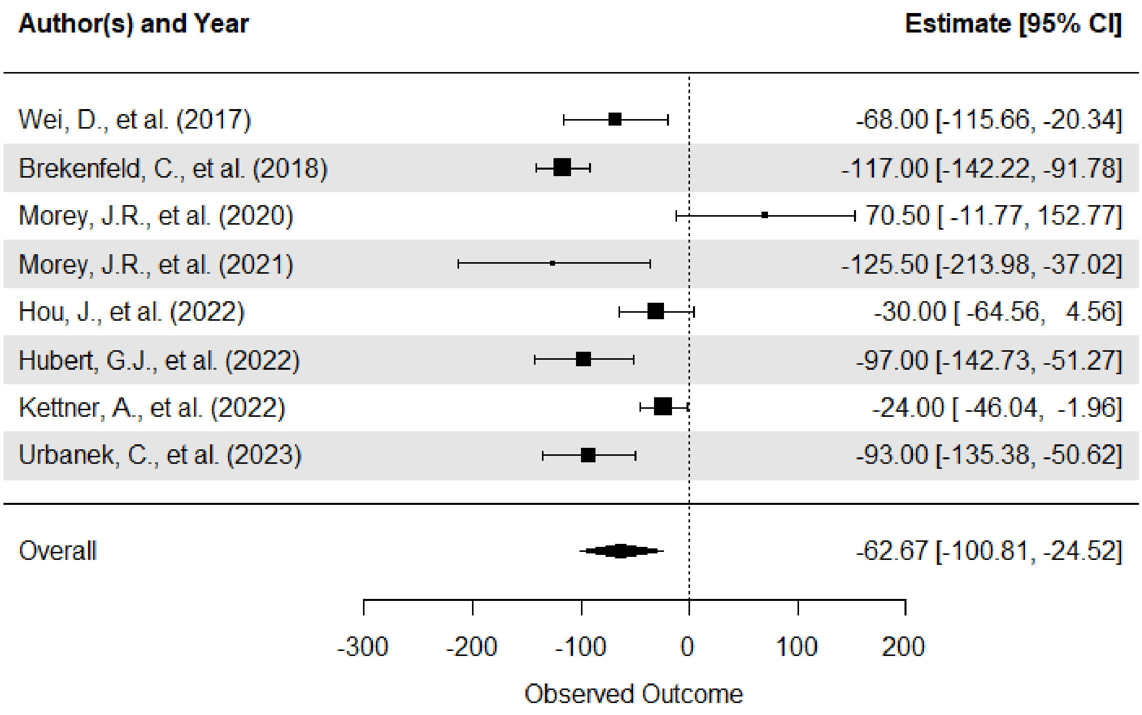

Supplement: Supplementary file 1 — Data S1 Supplemental Methods Tables S1–S3 Figure S1 [file JAH3-13-e031906-s001.pdf]
